# The Endoplasmic Reticulum Stress Response in Neuroprogressive Diseases: Emerging Pathophysiological Role and Translational Implications

**DOI:** 10.1007/s12035-018-1028-6

**Published:** 2018-03-29

**Authors:** Gerwyn Morris, Basant K. Puri, Ken Walder, Michael Berk, Brendon Stubbs, Michael Maes, André F. Carvalho

**Affiliations:** 1 Tir Na Nog, Bryn Road seaside 87, Llanelli, Wales SA15 2LW UK; 2 IMPACT Strategic Research Centre, School of Medicine, Deakin University, Geelong, Australia; 3 Department of Medicine, Imperial College London, Hammersmith Hospital, London, England W12 0HS UK; 4 The Centre for Molecular and Medical Research, School of Medicine, Deakin University, P.O. Box 291, Geelong, 3220 Australia; 5 Department of Psychiatry, University of Melbourne, Melbourne, Australia; 6 Orygen, the National Centre of Excellence in Youth Mental Health, Parkville, Australia; 7 Centre for Youth Mental Health, University of Melbourne, Melbourne, Australia; 8 Florey Institute for Neuroscience and Mental Health, Melbourne, Australia; 9 Physiotherapy Department, South London and Maudsley NHS Foundation Trust, London, UK; 10 Health Service and Population Research Department, Institute of Psychiatry, Psychology and Neuroscience, King’s College London, London, UK; 11 Faculty of Health, Social Care and Education, Anglia Ruskin University, Chelmsford, UK; 12 Department of Psychiatry, Chulalongkorn University, Bangkok, Thailand; 13 Department of Psychiatry, Faculty of Medicine, University of Toronto, Toronto, ON Canada; 14 Centre for Addiction & Mental Health (CAMH), Toronto, ON Canada

**Keywords:** Neurodegeneration, Neuroprogression, Unfolded protein response, Mood disorders, Endoplasmic reticulum stress, Molecular neurobiology

## Abstract

The endoplasmic reticulum (ER) is the main cellular organelle involved in protein synthesis, assembly and secretion. Accumulating evidence shows that across several neurodegenerative and neuroprogressive diseases, ER stress ensues, which is accompanied by over-activation of the unfolded protein response (UPR). Although the UPR could initially serve adaptive purposes in conditions associated with higher cellular demands and after exposure to a range of pathophysiological insults, over time the UPR may become detrimental, thus contributing to neuroprogression. Herein, we propose that immune-inflammatory, neuro-oxidative, neuro-nitrosative, as well as mitochondrial pathways may reciprocally interact with aberrations in UPR pathways. Furthermore, ER stress may contribute to a deregulation in calcium homoeostasis. The common denominator of these pathways is a decrease in neuronal resilience, synaptic dysfunction and even cell death. This review also discusses how mechanisms related to ER stress could be explored as a source for novel therapeutic targets for neurodegenerative and neuroprogressive diseases. The design of randomised controlled trials testing compounds that target aberrant UPR-related pathways within the emerging framework of precision psychiatry is warranted.

## Introduction

The endoplasmic reticulum (ER) is a cell organelle that plays an indispensable role in protein synthesis, folding and sorting, as well as the delivery of proteins to their ultimate cellular destination. This role is facilitated by the presence of a multitude of chaperone proteins capable of binding to hydrophobic areas of newly synthesised, but as yet unfolded, proteins to facilitate optimal protein folding and prevent protein–protein aggregation. Under physiological conditions, protein folding and function are also facilitated by *N*-linked glycosylation and the formation of disulphide bonds by reaction mechanisms favoured by the highly oxidative environment of the ER [1, 2].

**Fig. 1:**
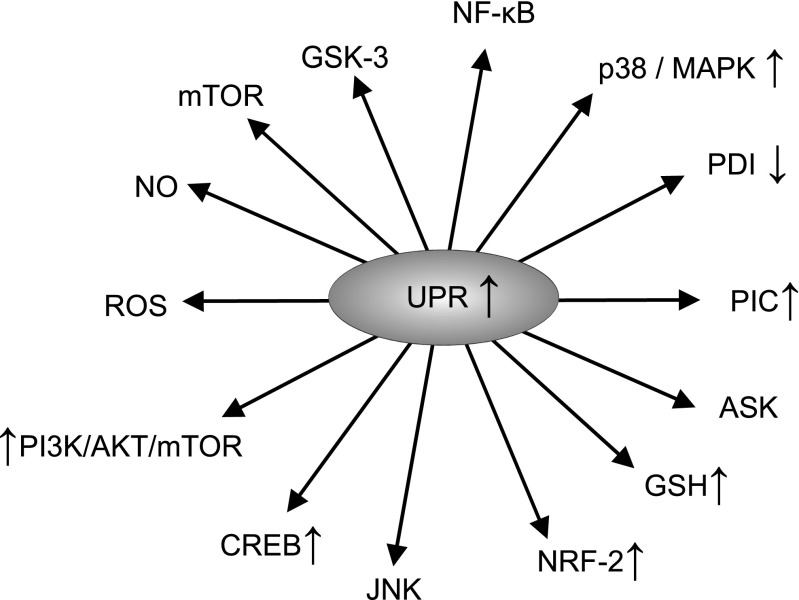
Effects of the upregulation of the UPR

However, in pathophysiological circumstances, the accumulation of misfolded or unfolded proteins may ensue [2, 3]. Several mechanisms may contribute to the accumulation of unfolded proteins, including an excessive biosynthesis of reactive oxygen species (ROS), a lowered efficiency of cellular anti-oxidant defences [2, 4], as well as disturbances in calcium homoeostasis [2, 3]. In addition, in diseases like amyloidosis and Huntington’s disease the accumulation of misfolded proteins appears to be a pivotal pathophysiological event. In such circumstances, the ER initially elicits an adaptive or protective response described as the unfolded protein response (UPR) aimed at restoring homoeostasis within the organelle and the cell through the re-establishment of protein homeostasis [5–7]. Nevertheless, in some pathophysiological situations, the homeostatic capacity of the ER and the UPR may not meet cellular demands and may even become detrimental (vide infra), a condition referred to as ER stress. While severe and prolonged ER stress may trigger apoptotic cell death [8, 9], there is an accumulating body of evidence supporting the proposition that sub-lethal ER stress and the consequent chronic upregulation of the UPR are involved in the pathogenesis and pathophysiology of several diseases [10–12]. Figure 1 summarises the effects of upregulation of the UPR.

Exemplars of such illnesses include Alzheimer’s disease [13, 14], Parkinson’s disease [15, 16], multiple sclerosis [17, 18] and amyotrophic lateral sclerosis [19, 20]. More recently, a putative role of ER stress for psychiatric disorders in which neuroprogression may occur, including bipolar disorder [12, 21, 22], major depressive disorder [23, 24] and schizophrenia, [25] has been disputed. It is noteworthy that the chronic upregulation of the UPR may lead to the development of chronic inflammation [26, 27], oxidative stress [11, 28, 29] and multiple dimensions of mitochondrial dysfunction [30–33] and that these elements appear to be shared factors involved in the pathogenesis and pathophysiology of neurodegenerative and neuroprogressive disease, although disease-specific elements also seem to be involved [34–39]. There is also some evidence to suggest that the detrimental effects of ER stress and chronic UPR upregulation could be “druggable” and hence inhibition of pathways involved in the UPR may confer neuroprotection. For example, there are reports demonstrating that inhibition of ER stress pathways could protect against neuronal injury [40–42].

Thus, this review has two overarching aims: first, to detail putative pathways whereby activation of the UPR may instigate or exacerbate chronic inflammation, oxidative/nitrosative stress and multiple dimensions of mitochondrial dysfunction that are observed across neuroprogressive illnesses and, second, to examine therapeutic options targeting ER stress and the UPR as novel neurotherapeutic targets for neuroprogressive diseases. Initially, processes stemming from ER stress and UPR activation which may lead to the initiation or exacerbation of chronic neuroinflammation will be critically examined before moving on to a consideration of putative pathways leading to the initiation or exacerbation of oxidative and nitrosative stress, and multiple dimensions of mitochondrial dysfunction.

## ER Stress, Activation of the UPR and the Development of Chronic Inflammation

### Processes Involved in the Activation of the UPR

During the UPR, a triad of ER transmembrane protein receptors referred to as protein kinase RNA-like endoplasmic reticulum kinase (PERK), inositol-requiring enzyme 1α (IRE1α) and activating transcription factor 6 (ATF6), whose activity is negatively regulated by the master ER chaperone GRP78, act as sensors to detect misfolded/mutant proteins [43, 44]. However, in an environment of ER stress, GRP78 binds to the exposed hydrophobic domains of unfolded or misfolded proteins leading to their dissociation from PERK, ATF6 and IRE1α, thus activating these ER signalling pathways [43]. Once activated, each of these receptors may undergo oligomerisation and other conformational changes, thus inducing highly specific downstream signalling cascades [44, 45].

### Activation of PERK and the Development of Chronic Inflammation

PERK phosphorylates eukaryotic translation initiation factor-2α (eIF2α) leading to an inhibition of general protein translation and promotion of the preferential translation of transcription factor ATF4 [7, 46]. ATF4 in turn translocates to the nucleus whereupon it induces the transcription of additional UPR target genes and, in an environment of extreme ER stress, ATF4 targets the promoter of the gene that encodes the transcription factor CHOP, which plays a major role in the instigation of apoptotic cell death [47] (see [48] for a review).

Activation of PERK leads to upregulation of the JAK1/STAT3 signalling axis and subsequent increments in the transcription and translation of IL-6 and oncostatin, thus forming a feed-forward loop driving escalating levels of inflammation [49]. It is noteworthy that activation of PERK in astrocytes, and subsequent paracrine activation of microglia, is now recognised as a relevant mechanism in the initiation and perpetuation of neuroinflammation [49]. PERK activation leads to phosphorylation of eIF2α, which also suppresses the translation of IκB, resulting in translocation of the cytosolic transcription factor NF-κB to the nucleus, whereupon it may induce the expression of genes involved in instigating and regulating inflammatory pathways [50]. Furthermore, PERK may also regulate cellular redox homoeostasis via the activation of nuclear factor erythroid 2-related factor 2 (Nrf2) and the subsequent upregulation of reduced glutathione [51–53]. It is also noteworthy that PERK-activated ATF4 also regulates the cellular redox state and may also act independently of PERK to induce the production of pro-inflammatory cytokines [50].

### Activation of ATF6 and the Development of Chronic Inflammation

Upregulation of monomeric ATF6 also exerts a range of complex, broadly pro-inflammatory effects via the upregulation of NF-κB via mechanisms involving activation of the CREB and PI3K/Akt/mTOR signalling pathways [50, 54]. The upregulation of this UPR pathway also exerts direct effects on inflammation via the upregulation of toll-like receptor activity on macrophages [55].

### Activation of IRE1α and the Development of Chronic Inflammation

**Fig. 2:**
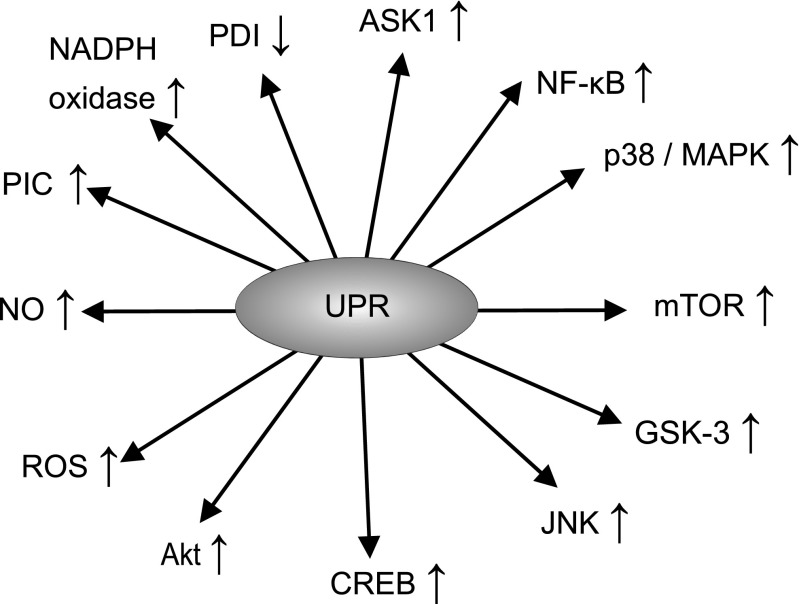
Actions of the UPR

IRE1α functions both as a kinase and as an endonuclease, which is activated via a process of oligomerisation in the absence of GRP78 inhibition. Evidence suggests that this enzyme could play a major role in regulating the splicing of several mRNAs and its activity is an indispensable player in the translation and activation of transcription factor X-box binding protein-1 (XBP-1) [56, 57]. XBP-1, in turn, increases the transcription of several UPR target genes including the one encoding GRP78 [58, 59]. The activated IRE1α can also form a multiprotein complex with apoptosis signal-regulating kinase 1 (ASK1), resulting in the upregulation of various intracellular signalling systems such as c-Jun N-terminal kinase (JNK) [60], p38/MAPK [61, 62], NF-κB [63, 64], glycogen synthase kinase 3 (GSK-3) [65, 66], mammalian target of rapamycin (mTOR) [67, 68] and the phosphatidylinositol 3-kinase/protein kinase B/mTOR (PI3K/AKT/mTOR) pathway [69–71]. These pathways also play a major role in determining the balance between cell survival and cell death, generally promoting cell survival in an environment of chronic oxidative stress. Yet it is important to note that their effects on cell survival are pleiotropic, and activation of these pathways may also drive cellular death in other circumstances, particularly when ER stress is severe [72, 73]. The net effect of these signalling systems is somewhat unpredictable as they engage in a complex pattern of mutual cross-talk with the UPR and each other, and their relative activities appear to influence the balance between cell proliferation and cell death [74–76]. Figure 2 illustrates the actions of the UPR.

For example, the UPR activates GSK-3, possibly via a route involving increased autophagy of the inactive kinase phosphorylated at serine 219 [65]. This kinase in turn also appears to play a role in the regulation of the UPR by influencing the phosphorylation status of CHOP and caspase-3 [66, 77]. There is also evidence of a bidirectional feedback between UPR activity and levels of mTOR signalling [78–80]. Similarly, the activation of NF-κB by the UPR also acts to reduce ER stress by accelerating the clearance of misfolded proteins via the modulation of autophagic activity [72]. Readers interested in a detailed consideration of the mechanisms enabling and regulating such “cross-talk”, and how such communication leads to variations in biochemical and immunological profiles over time, are referred to previous scholarly reviews [81, 82]. Importantly, from the perspective of the aims of this paper, changes in the activity of p38/MAPK, JNK, NF-κB, mTOR, GSK-3 and PI3K/AKT have pivotal roles in instigating and/or modulating inflammatory and immune pathways and the activity of peripheral mononuclear blood cells such as macrophages [83–87].

Several research teams have adduced data demonstrating that p38/MAPK is a major player in the promotion and regulation of inflammatory and immune responses in general and that the upregulation of p38/MAPK is a pivotal driver of pro-inflammatory cytokine transcription and translation [88]. Phosphorylation of NF-κB and a range of other transcription factors, such as myocyte enhancer factor-2 (MEF-2), as a result of upregulated p38/MAPK activity induces transcriptional activation of tumour necrosis factor-α (TNF-α), IL-6 and other pro-inflammatory cytokines [83, 89]. Similarly, there is copious evidence that activation of JNK signalling plays a major role in cytokine production and the subsequent development of inflammation [84, 90].

The NF-κB pathway also regulates the production of pro-inflammatory cytokines and several other processes driving the inflammatory response, such as leukocyte recruitment and the survival of peripheral mononuclear blood cells, which are important contributors to the inflammatory response [91, 92]. Furthermore, the complex bidirectional signalling between pro-inflammatory cytokines (notably TNF-α) allows for the development of a self-amplifying inflammatory response [93, 94]. However, it should be noted that the anti-apoptotic activity of NF-κB may protect cells against the ravages of inflammation and in certain circumstances the pro-apoptotic properties of this signalling system can also contribute to the resolution of inflammation by contributing to the immunologically silent destruction of infiltrating leucocytes and macrophages [91].

The activity of GSK-3 influences the balance between the production of pro- and anti-inflammatory cytokines, T cell differentiation, toll-like receptor responses and the proliferation and activity of transcription factors which are known to play a regulatory role in the duration and magnitude of the immune response, such as signal transducer and activator of transcription (STAT), nuclear factor of activated T cells (NFAT), T-box transcription factor (Tbet) and NF-κB [95–97]. A recent review further illustrates the immunoregulatory role of GSK [98]. Much of this regulatory activity occurs in concert with mTOR and PI3K/AKT pathways [99]. These interactions are complex but are essentially connected with the role of mTOR as a metabolic sensor and its capacity to integrate metabolic and immune processes, thereby regulating the activation and proliferation of T cells, B cells and antigen presenting cells. Readers interested in a detailed consideration of the processes involved are invited to consult the work of Powell et al. [100] and Weichhart et al. [85]. It should also be noted that the PI3K/AKT/mTOR signalling axis has a broadly restraining effect on the development of chronic inflammation by limiting the production of type 1 interferons, while increasing the production of IL-10, and hence, its downregulation during chronic UPR activation may contribute to the development and perpetuation of an inflammatory state [86].

Given the above data, accumulating evidence supporting an association between the chronic upregulation of the UPR and the development of chronic inflammation is perhaps unsurprising [26, 65, 70, 101]. It is also noteworthy that the chronic upregulation of pro-inflammatory cytokines in tandem with upregulation of NF-κB and p38/MAPK may enhance the biosynthesis of ROS and nitric oxide (NO), and thus may promote or otherwise aggravate oxidative and nitrosative stress [87, 102–104], and hence provides a mechanism for the development of chronic oxidative stress accompanying acute or chronic upregulation of the UPR [11, 28, 105]. Moreover, the complex interplay of NF-κB, p38/MAPK and ROS may lead to a self-amplifying pattern of redox dyshomoeostasis [106–108]. However, there are a number of other mechanisms which may also contribute to the development of oxidative and nitrosative stress following ER stress and over-activation of the UPR which seem underdiscussed, and we will now turn to a consideration of these factors.

## UPR Activation and the Development of Oxidative and Nitrosative Stress

### Mechanisms Involved in the Development of Chronic Oxidative and Nitrosative Stress

ER stress and the subsequent activation of the UPR may lead to an increased production of ROS and subsequently to oxidative stress via a number of mechanisms other than the upregulation of MAPK and NF-κB [105]. Such mechanisms involve an upregulation of protein disulphide isomerase (PDI) resulting in the activation of NADPH oxidase isomers, notably NOX-2 and NOX-4 [109], and the upregulation of oxidative protein folding in the ER, which rivals mitochondrial respiration as a source of cellular ROS [110, 111]. Other factors involved in the development of oxidative stress in such circumstances include the oxidation of reduced glutathione (GSH), an increased *S*-nitrosylation of proteins and an increase in Ca^2+^ efflux from the ER into the mitochondria [28, 112].

### Upregulation of PDI and Activation of NADPH Oxidase Isoforms

The development of ER stress and activation of the UPR may lead to upregulation of PDI [113–116]. This is an important event in the context of the development of oxidative stress, as PDI is associated with NOX isoforms and acts as a redox-sensitive protein which regulates their activation [10, 109, 117]. The change in cellular redox status “sensed” by PDI thus may activate NOX-2 and NOX-4 [10, 118, 119], leading to the production of superoxide ions [120, 121].

The effects of PDI in activating NOX enzymes appear to be of pathophysiological relevance since these enzymes are a major source of ROS in several cell types [122, 123], and ROS production by NOX isoforms may even exceed mitochondria as the prime source of ROS in some cell types [124]. However, while ROS production within mitochondria stems from the integral architecture and membrane organisation, NOX signalling is dependent on multiple protein interactions and post-translational modifications leading to the assembly of a functional NOX complex and the subsequent trafficking to specific subcellular locations [122, 123]. The assembly of subunits and the translocation NOX enzymes to sites of activity appears to be met by the chaperone rather than the isomerase activity of PDI via hydrophobic rather than electrostatic or covalent associations [109, 125]. Nevertheless, both the chaperone and isomerase activities of PDI are required to fulfil its role in the oxidative folding of proteins within the ER [126].

### Upregulation of PDI and Increased Rate of ROS and RNS Production from Oxidative Protein Folding

The ER contains numerous molecules whose task is to ensure that proteins secreted from the organelle have acquired the prerequisite post-translational modifications and the correct conformation [127]. One important process involved in ensuring optimal protein folding is the acquisition of disulphide bonds. The interaction between PDI and oxidoreductin-1α (Ero1α) is probably the most important vehicle for oxidative protein folding in the ER [128, 129]. Hence PDI has the capacity to supply, isomerise or, in some circumstances, reduce disulphide bonds in target proteins [130], while its activity is dependent upon the existence of two distinct remote active sites which are directly or indirectly oxidised by Ero1α to form disulphide bridges [130–132]. Such oxidation provokes a conformational change allowing for the entry of unfolded protein substrates in the reduced state [133, 134]. Once in situ, key thiol groups on these proteins are oxidised to form disulphide bridges, resulting in the reduction of PDI; these target proteins then “receive” disulphide bonds from PDI; Ero1α then re-oxidises the reduced PDI and transfers electrons from the reduced PDI to molecular oxygen, which is subsequently reduced to hydrogen peroxide (H_2_O_2_) [135, 136], thus resulting in the re-oxidation of the oxidoreductase [128, 137]. The capacity of Ero1α to reduce molecular oxygen is dependent on the existence of a helical structure containing flavin adenine dinucleotide (FAD) sealed by a disulphide bridge between Cys(208)-Cys(241). This “seal” may be disrupted via the formation of a mixed disulphide bridge between PDI and one of these cysteines, which underpins the capacity of this chaperone to regulate the activity of its co-oxidoreductase [135, 136]. In addition, Ero1α activity in the ER is upregulated by the UPR and hence H_2_O_2_ levels may increase as a result of ER stress [135, 136]. Initially, such upregulation may have an adaptive purpose as the glutathione peroxidase isoform GPx7 may utilise H_2_O_2_ to accelerate the oxidative folding of substrates in vivo [65]. Briefly, evidence suggests that H_2_O_2_ oxidises the Cys57 residue of GPx7 to produce sulfonic acid, which in turn may react with its Cys86 to form a disulphide bond. Both the disulphide and the sulfonic acid forms of GPx7 may oxidise PDI to catalyse oxidative folding [138]. However, the accumulation of ROS and reactive nitrogen species (RNS) following activation of the ER [139, 140] leads to *S*-nitrosylation and the subsequent inactivation of PDI, thus leading to a loss of its chaperone and isomerase activities [140–142]. This loss of activity may have meaningful pathophysiological consequences; the accumulation of misfolded proteins within the ER may further enhance the UPR, leading to self-amplifying increases in inflammation as well as oxidative and nitrosative stress [143, 144]. Importantly, such increases in ROS and RNS may also promote ER stress, which leads to an increase in Ca^2+^ efflux from the ER into mitochondria [28, 112] which is enabled by tubular channels tethering the organelles described as mitochondrial associated molecular membranes (MAMs) [5, 145]; an increase in Ca^2+^ within the mitochondria may ultimately lead to the development of multiple dimensions of mitochondrial dysfunction as discussed below.

## ER Stress, UPR Activation and Mitochondrial Dysfunction

### Initial Increase in Mitochondrial Respiration

The ER and mitochondria are physically connected by highly specialised structures referred to as MAMs. These molecules act as a conduit for the exchange of proteins, lipids, a range of metabolites, various signalling molecules and most importantly Ca^2+^, and this complex cascade of events appears to influence the balance between cell death and survival [5, 146]. The architecture of a MAM is highly complex and contains a wide array of structural, functional and regulatory proteins, such as the GTPase activating protein for Rab32 [147, 148]. The inositol trisphosphate receptor (IP3R) and the voltage-dependent anion channels (VDACs) are among the most important molecules for enabling and regulating ER–mitochondria Ca^2+^ transfer, and are located in the ER and mitochondrial sides of MAMs, respectively, and may complex with the chaperone GRP75, thus forming a channel connecting the two organelles and enabling mutual exchange between membrane and luminal components [149, 150]. Mitofusin 2 (Mfn2) is another important protein present on the ER and mitochondrial surfaces, which plays an indispensable role in ER–mitochondria tethering as well as in the modulation of inter-mitochondrial contacts [5, 151, 152]. The composition of MAMs adapts in response to multiple internal and external stimuli [153, 154], while the formation or dissolution of contact areas between mitochondria and the ER is further regulated by other aspects of organelle dynamics [5, 148, 154]. Importantly, in the adaptive phase of ER stress, there is an increased number of physical contacts between the ER and mitochondrial networks at the perinuclear regions enabling increased transfer of Ca^2+^ from the ER into the mitochondria [5, 43, 146, 155].

An increase in Ca^2+^ uptake by the mitochondria may increase transmembrane potential and ATP production aimed at promoting cellular survival as part of an adaptive response to ER stress [156]. Such an increase in energy production is accompanied by increases in the production of mitochondrial proteases such as LON, which are induced by the activation of the PERK pathway, which in turn regulates the structural integrity and assembly of cytochrome *c* oxidase (COX) [157, 158]. In this scenario, elevated expression of LON protease may increase mitochondrial performance by stimulating the assembly and increasing stabilisation of COX II [157, 158]. However, elevated calcium levels may also increase the production of ATP and ROS [159–161], leading to the activation of mitochondrial nitric oxide synthase (mtNOS) [162, 163], and the production of NO, leading to the inhibition of mitochondrial function via a number of direct and indirect mechanisms including the reversible *S*-nitrosylation of key structural and functional mitochondrial proteins and enzymes [163–165].

**Fig. 3:**
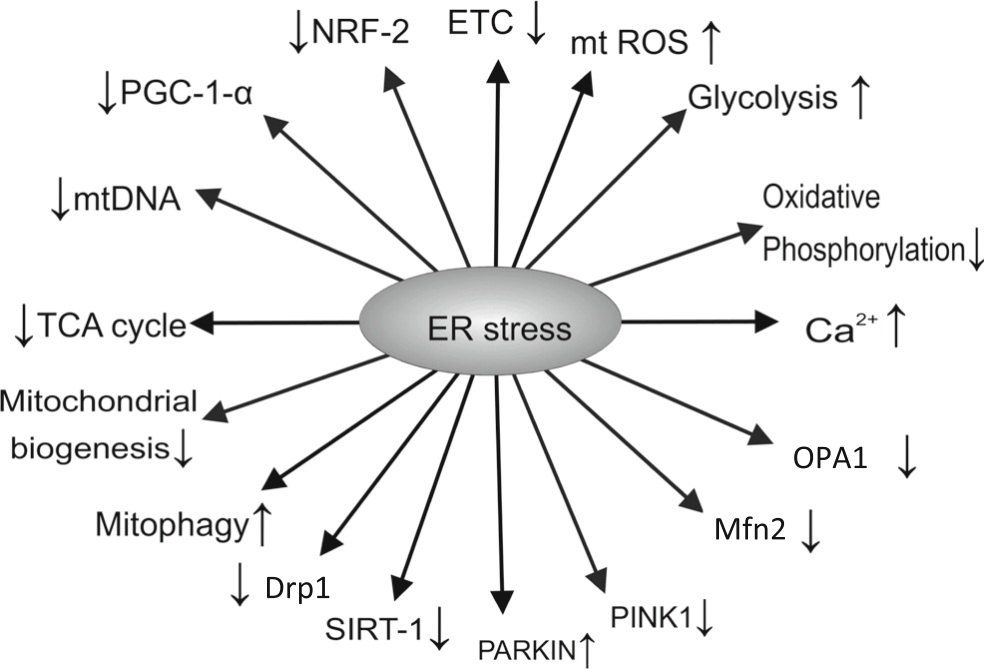
Effects of ER stress

Figure 3 summarises the effects of ER stress.

### Elevated Levels of NO and Mitochondrial Function

The nitrosylation of mitochondrial structural proteins and enzymes may play a major role in the redox-based regulation of mitochondrial respiration [166, 167]. While nitrosylation in response to modest increases in NO levels may initially act as a defence mechanism aimed at maintaining protein structure and function [168–170], further increases in this RNS may lead to the inhibitory nitrosylation of crucial functional enzymes such as complex I of the electron transport chain [165, 171]. Furthermore, the inhibition of complex I by *S*-nitrosylation is another initially cytoprotective response, which also leads to decreased ATP production and defects in energy homoeostasis over time [169, 170]. Persistently elevated cellular concentrations of NO may also lead to the inhibitory nitrosylation of crucial functional cysteine thiols of COX and complex II of the electron transport chain, thus leading to chronically suppressed activity of the former and transiently reduced activity of the latter [172, 173]. Such inhibition may ultimately impair oxidative phosphorylation and hence decrease ATP production and GSH levels within the organelle [173–175]. Furthermore, the prolonged inhibition of COX activity also provokes an increase in ATP production via glycolysis in a wide range of cell types as a defensive response aimed at preventing apoptosis or necrosis [176–178]. Importantly, the inhibition of complex III and complex IV by *S*-nitrosylation may further increase the production of ROS [179, 180], which combined with reduced ATP generation may contribute to the release Ca^2+^ from the ER [181–183], which may further decrease the biosynthesis of ATP and also increase the generation of ROS in a positive feedback loop [110, 184]. This process is of relevance, as an increased production of ROS may increase the misfolding of mitochondrial proteins, which coupled with impaired oxidative phosphorylation and ATP production may trigger another response aimed at restoring mitochondrial homoeostasis, namely the mitochondrial unfolded protein response (mtUPR) [185–188]. Thus, in the section below, we also discuss the putative pathophysiological relevance of the mtUPR.

### Impaired Mitochondrial Performance Following Activation of the mtUPR

The mtUPR is a multidimensional transcriptional response initiated and maintained by retrograde mitochondrial-to-nuclear signalling following increases in protein misfolding in the mitochondrial matrix and inner membrane space and/or decreased efficiency of protein importation into mitochondria aimed at restoring mitochondrial function and preventing organellar death [189–192].

The initiation of the mtUPR is mediated by sensory quality control proteases with LON or ClpCP being the prime activators in the matrix [191] and the mitochondrial serine protease HTRA2 playing the same role at the inner membrane space [193–195]. Interestingly, the initial upregulation of HTRA2 is provoked by an overproduction of ROS and the subsequent phosphorylation of Akt, which in turn activates the oestrogen receptor in the outer mitochondrial membrane leading to upregulation of the transcription factor nuclear respiratory factor 1 (NRF-1) and ultimately leading to increased mitochondrial production of HTRA-2 [193]. This is an illustrative example of retrograde mitochondrion to nucleus signalling and is similar in principle to the retrograde ER to nucleus signalling that facilitates the UPR response (reviewed in [196]). Another example involves the upregulation of CHOP, which is an indispensable player in the regulation of mitochondrial proteases and chaperones, in an attempt to restore intra-mitochondrial protein folding homoeostasis [197, 198]. However, despite recent evidence suggesting that signals of protein unfolding within mitochondria are transduced to the nucleus via activation of c-Jun, JNK and the activator protein 1 (AP-1) [64, 191], thus sharing some characteristics with the ER UPR, the precise details underpinning this mechanism remain to be elucidated [190]. It should also be noted that while there is some evidence that the factors involved in initiating and regulating the mtUPR are similar in principle to those that regulate the ER UPR, there is another regulatory mechanism governing the mtUPR, namely decreased mitochondrial import efficiency, which is unique to the mtUPR, and some background information is required to understand its genesis and implications.

The vast majority of mitochondrial proteins originate from nuclear DNA and hence must be recruited to the mitochondria and thereafter imported. In most circumstances, this recruitment is initially achieved via the mitochondrial targeting sequence (MTS) [199]. Once in situ at the outer mitochondrial membrane (OMM), the protein is directed via a myriad of regulatory processes to either the OMM, the intermembrane space, the inner mitochondrial membrane (IMM) or the matrix. Importantly, in order to enter the matrix, the protein must cross the IMM via the translocase of inner membrane complex (TIM), which requires the optimal activity of chaperones located at the matrix as well as physiological tricarboxylic acid (TCA) cycle and oxidative phosphorylation activities [199, 200]. Hence, mitochondrial protein import efficiency may provide a proxy measure of diverse aspects of mitochondrial performance [191, 201, 202]. Importantly, a lowered import of proteins into the mitochondria leads to the accumulation in the cytoplasm of proteins normally destined for the organelle [64, 203]. Most such proteins are detected and targeted for proteasomal degradation [204, 205]. However, in lower animals, at least one mitochondrial protein, the transcription factor ATFS-1, which regulates the mtUPR in the worm *Caenorhabditis elegans*, has both a MTS, which enables its mitochondrial import in normal physiological conditions, and a nuclear localisation sequence (NLS), which enables its translocation to the nucleus in conditions of mitochondrial stress whereupon it activates the mtUPR [187]. There are excellent reviews detailing this process [190, 203]. Notwithstanding that evidence of such a transcription factor in mammals is lacking, Fiorese et al. [206] have recently reported the existence of ATF5 in mammalian cells which is regulated similarly to ATFS-1 and may induce a similar transcriptional response.

The mtUPR is activated by a range of stressors other than the presence of unfolded proteins, which may lead to a decrease in mitochondrial protein import efficiency. In addition to the presence of heavy metals or other substances acting as DNA adducts, contaminants in sulphide bonds, or otherwise, such stressors include a depletion of mtDNA [186, 207], high levels of ROS [185, 186], mitochondrial ribosome impairment [208, 209], inhibition of mitochondrial proteases and chaperones [186], impaired oxidative phosphorylation and ATP production [187, 188] and abnormally high glucose consumption indicating a switch to the glycolytic pathway as a source of energy generation [210].

It should be stressed that while one facet of the mtUPR involves the upregulation of genes aimed at increasing mitochondrial proteases as well as chaperones, thereby promoting protein homoeostasis within the mitochondrial protein folding environment [186, 211], another facet includes changes in the transcription patterns of genes governing cellular metabolism [190]. In particular, the mtUPR may increase the expression of genes governing the rate of glycolysis and the catabolism of amino acids with a concomitant suppression of genes enabling the optimal performance of the TCA cycle and oxidative phosphorylation [212, 213].

Therefore, while aimed at relieving mitochondrial stress and ensuring cellular survival, the over- or chronic activation of the mtUPR may also compromise mitochondrial function and oxidative phosphorylation, thus favouring a switch to aerobic glycolysis as the predominant source of ATP [212, 213]. These changes in cellular metabolism provoked by the activation of the mtUPR could be of interest given data demonstrating that such a response may be regulated by NAD^+^ and sirtuin (SIRT) deacetylases [193, 209, 213], which are capable of sensing and stimulating metabolic activity by increasing mitochondrial performance via a number of different routes (reviewed by Morris et al. [214]). Importantly, SIRT-1 is inactivated in an environment of nitro-oxidative stress [215] and such inactivation may up-regulate NF-κB [215]. Hence, in a cellular environment of chronic oxidative stress the normal compensatory response to impaired mitochondrial function is negated and the switch to aerobic glycolysis via NF-κB upregulation is preferentially operating [216].

Furthermore, the progressive decline in mitochondrial ATP production and mitochondrial membrane potential in such circumstances coupled with an increase in aerobic glycolysis can activate another very specific mitochondrial quality control mechanism involving retrograde mitochondrion to nucleus signalling referred to as mitophagy [217, 218]. Such a physiologically abnormal elevation in the rate of mitophagy has adverse bioenergetic consequences as this process appears to play a relevant role in the regulation of energy homoeostasis and mitochondrial dynamics [219, 220]. It is also noteworthy that ER stress and the UPR accompanied by increased levels of Ca^2+^ and ROS can also exert detrimental effects on multiple regulatory processes governing mitochondrial dynamics directly [221–223].

**Fig. 4:**
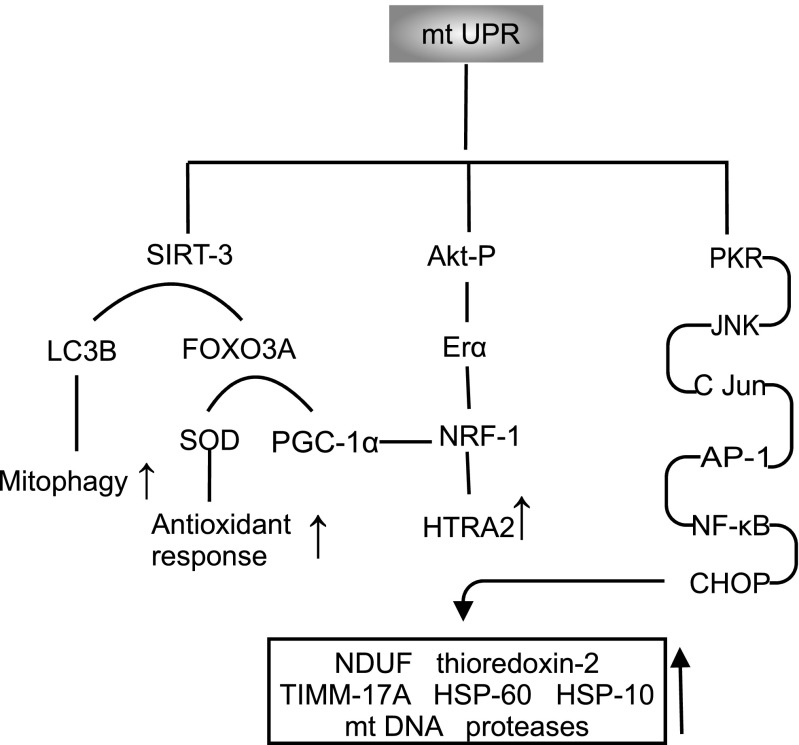
Key reactions and pathways associated with the mtUPR

Key reactions and pathways associated with the mtUPR are summarised in Fig. 4.

## UPR Activation and Impaired Mitochondrial Dynamics

### Background

Accumulating evidence indicates that the balance of activity between pathways regulating mitophagy and those regulating mitochondrial dynamics (mitochondrial biogenesis, fusion, fission and motility) may influence mitochondrial mass, morphology and function and thus the cellular capacity to generate energy and to adjust energy production in the face of changing metabolic demands [219, 224, 225]. In particular, changes in mitochondrial dynamics enable these organelles to maintain a balance between energy production and changes in energy demand by generating highly fused networks of mitochondria or otherwise favouring the formation of more discrete and isolated organelles [226–228]. In addition, pathways and proteins governing mitochondrial dynamics may regulate energy supply and distribution at both the whole organism and cellular levels [229]. Therefore, the targeted manipulation of these processes may open a relevant therapeutic perspective for neuroprogressive disorders. Therefore, facets of mitochondrial dynamics as well as the pathophysiological influence of the chronic upregulation of the UPR on these processes will now be discussed as the final mechanistic section of this paper.

### ER Stress and UPR Activation as a Source of Impaired Mitochondrial Mitophagy

Mitophagy is mediated by the cooperative action of the two proteins parkin and PINK. There are excellent reviews detailing the processes involved in the delivery and regulation of mitophagy [203, 219, 225]. Therefore, we provide a brief description of this process in order to explain the putative adverse effects of the UPR upon mitophagy.

PINK1 is a serine/threonine kinase that possesses an N-terminal mitochondria-targeting signal [230] enabling anchorage at the IMM. Under physiological conditions, PINK1 is imported into the mitochondria via TIM and translocase of outer membrane (TOM) protein complexes and is continuously degraded by PARL and matrix processing peptidase [230, 231]. However, following the accumulation of unfolded proteins and/or membrane depolarisation, mitochondrial import efficiency falls and therefore the import of PINK1 to the IMM is compromised [191, 201, 202]. Following such inhibition, the enzyme accumulates at the OMM and forms a large 700-kDa complex with TOM before undergoing activation via autophosphorylation at two serine residues [232, 233]. This activation results in the recruitment of inactive cytosolic parkin onto damaged mitochondria, whereupon the molecule is activated via PINK1-mediated phosphorylation [234]. Following activation, parkin ubiquinates a myriad of mitochondrial substrates as well as itself [235]. These ubiquitinated residues in turn undergo phosphorylation, which is affected by PINK1, thereby triggering further cycles of parkin recruitment in a feed-forward amplification loop [236, 237]. It should be noted that while this process is a prerequisite for the development of mitophagy, it is not sufficient in itself to precipitate the phenomenon, and other mechanisms also appear to play a role. Readers interested in a detailed account of such mechanisms are invited to consult previous work [238–240]. Moreover, mitophagy is also regulated by other processes governing mitochondrial dynamics [241–243], and an imbalance between mitophagy and mitochondrial biogenesis stemming from the activation of the UPR is now thought to play a relevant role in the pathophysiology of several neurodegenerative and neuroprogressive illnesses [225]. This is unsurprising given that the complex cross-talk between these processes is an essential element in regulating cellular energy homoeostasis [244, 245]. Importantly, changes in the rate of mitophagy may deregulate mitochondrial biogenesis [219, 246], thus compromising cellular energy homoeostasis.

### UPR Activation and Impaired Mitochondrial Biogenesis

Under physiological conditions, mitochondrial biogenesis is regulated by a sophisticated interplay between the coactivator peroxisome proliferator-activated receptor gamma coactivator-1 alpha (PGC-1α) and the transcription factors NRF-2 and SIRT-1, which enable coupling between changes in cellular metabolism to changes in mitochondrial mass and number [34, 214, 247]. However, in an environment of ER stress and UPR activation, elevated levels of NF-κB, MAPK and PKB/Akt as well as higher NO signalling provoke an increase in PGC-1α, NRF-1, NRF-2 and SIRT-1, which in turn induce an increase in mitochondrial biogenesis as a putative adaptive (i.e. pro-survival) response [248, 249]. However, with increasing levels of inflammation, and increased levels of oxidative and nitrosative stress, the upregulation of PGC-1α is inhibited by TNF-α [104] and the activity of NRF-2, SIRT-1 and NF-κB may be inhibited by *S*-nitrosylation or over-oxidation of cysteine residues which normally enable their function [171, 214], ultimately leading to a chronic state of decreased mitochondrial biogenesis. The processes governing mitochondrial biogenesis and those governing mitochondrial fusion and fission also engage in a complex bidirectional cross-talk which also plays a role in regulating cellular energy homoeostasis [250], and hence, impaired mitochondrial biogenesis can provoke adverse changes in processes governing mitochondrial fusion and fission which also have the effect of dysregulating cellular energy generation. In addition, the molecular players generating inflammation and oxidative stress also lead to compromised activity of proteins and pathways regulating fusion and fission, which may lead to decreased energy production at cellular and whole organism levels.

## UPR Activation and Impaired Activity of Proteins and Processes Governing Fusion and Fission

### Background

Mitochondrial fusion and fission processes are regulated and enabled by dynamin family GTPases [251]. Readers interested in a detailed explanation of the mechanisms underpinning the actions of these molecular motors are referred to the work of Ferguson and De Camilli [252]. In mammals, the fusion of OMMs is mediated by Mfn1 and Mfn2, whereas the fusion of inner membranes is mediated via the protein optic atrophy 1 (OPA1) [151, 253–256]. We will focus on their role in mitochondrial respiration and how their activities may be compromised in an environment of ER stress and chronic activation of the UPR.

### Role of Mitofusins in Energy Production and Consequences of UPR Upregulation

Mfn2, and to a lesser extent Mfn1, plays pivotal roles in the regulation of mitochondrial respiration and energy homoeostasis [228, 257, 258]. This role is perhaps unsurprising given that Mfn2 is an indispensable player in tethering mitochondria and ER stress and enabling high fidelity and rapid calcium signalling cross-talk between the two organelles in environments of stress and changing metabolic demands for energy [259, 260]. While modulation of calcium signalling appears to be one element underpinning the capacity of Mfn2 to regulate mitochondrial respiration energy and adaptation to increased cellular demands for energy, other mechanisms are clearly involved. Such mechanisms involve the inhibition of ROS production and the regulation of glucose homoeostasis via mechanisms which are not related to effects on calcium signalling, although the precise details of such mechanisms remain to be fully delineated [257, 258]. Crucially, the capacity of this enzyme to adapt the production of ATP by hypothalamic neurones is a major factor in regulating whole body metabolism and whole body energy homoeostasis [257, 258]. In this context, it is of paramount importance that the activity of this enzyme may be inhibited in an environment of chronic inflammation and nitrosative stress. For example, MAPK upregulation suppresses Mfn2 activity [261] and there is some evidence that this enzyme is inhibited in an environment in which the production of pro-inflammatory mediators is elevated [257]. It is also of interest that the capacity of Mfn2 to stimulate mitochondrial function is dependent on the activation of the PI3K/Akt pathway.

### Role of OPA1 in Energy Generation and Consequences of UPR Upregulation

There is some evidence to suggest that processing of the mitochondrial dynamin-like GTPase OPA1 is the main regulatory element governing mitochondrial function by modulating IMM potential [262]. Several research teams investigating the effects of *OPA1* mutants have adduced evidence indicating that OPA1 activity is an important factor determining the existence of cellular mitochondria as highly fused networks or a myriad of fragmented organelles, which affect the supply of ATP produced by oxidative phosphorylation and influence the capacity to increase cellular energy output in the face of increased metabolic demands for energy as discussed above [263, 264]. These observations have been supported by recent in vitro data supplied by Kao and fellow workers who reported that inactivation of OPA1 results in the fragmentation of established mitochondrial networks as well as a reduction in oxygen consumption, uncoupling of oxidative phosphorylation to ATP production and a shift to aerobic glycolysis as the main mode of energy generation [265].

OPA1 has several other regulatory roles in mitochondrial function such as maintaining the integrity of the quaternary structures of electron transport chain enzymes and preventing depolarisation of the IMM. In addition, OPA1-dependent stabilisation and remodelling of mitochondrial cristae increases the efficiency of energy production by the electron transport chain, while also reducing the production of ROS [266]. OPA1-driven cristae remodelling is another essential factor enabling cells to meet energy production in the context of enhanced energy demands [267]. The importance of OPA1 in this domain is further emphasised by the existence of data demonstrating that its targeted inactivation leads to detrimental changes in crista morphology and reduces the stability and performance of the electron transport chain, thereby compromising oxidative phosphorylation and ATP production [268, 269].

Other roles include stabilising the association between cardiolipin and COX, thereby acting as an anti-apoptotic protein [266, 268–270]. Perhaps predictably, there is experimental evidence that *OPA1* transcription and translation are upregulated in an environment of chronic nitro-oxidative stress [271], which is also supported by data demonstrating that mitochondrial dynamics in general, and OPA1 levels in particular, appear to be under the control of the non-canonical NF-κB pathway [272]. In addition, evidence indicates that levels of this enzyme are elevated following activation of the Akt/mTOR pathway [273] and more indirectly by ROS and Ca^2+^ and by upregulation of PGC-1α [274]. The involvement of AKT and NF-κB in the regulation of OPA1 activity is particularly germane as both molecules are inactivated by *S*-nitrosylation in an environment of nitro-oxidative stress [275–278], and such inactivation may compromise the ability of mitochondria to cope with elevated cellular requirements for energy.

### The Role of Drp1 in Energy Generation and the Consequences of UPR Upregulation

The activity of the mitochondrial fission protein Drp1 is regulated by a plethora of factors such as Ca^2+^ concentrations, ROS levels and a range of post-translational modifications [279–281] (see [282] for a review). Chronically elevated levels of ROS and RNS lead to changes in Drp1 activity and/or rates of mitochondrial fission via a number of routes. One such route involves the inactivation of Drp1 by AMPK, whose activity is upregulated in an environment of chronically elevated ROS generation [214, 283, 284]. This is of importance as there is evidence that inhibition of this GTPase may disrupt mitochondrial networks, thus leading to adverse changes in organelle morphology accompanied by a reduction in Mfn1 and Mfn2 as well as a compromised proteolytic processing of OPA1 isoforms [285] and hence presents yet another route by which the chronic upregulation of the UPR could compromise energy generation. It is also noteworthy that *S*-nitrosylation of Drp1 in an environment of chronically upregulated nitro-oxidative stress could increase the rate of mitochondrial fission [286], thereby creating an imbalance between fusion and fission which may lead to detrimental net alterations in mitochondrial morphology and energy production [287–289]. The precise mechanisms underpinning such an increase in fission rates is still a matter of ongoing debate. However, it may not be a direct consequence of nitrosylation-induced increases in the enzymatic activity of Drp1 [290]. Lastly, mitophagy relies on a synergistic interplay between parkin and the dynamin family kinase Drp1, with the fission activity of the latter required to generate small mitochondria, thereby enabling efficient engulfment by autophagosomes [239, 291]. Hence, inhibition of this enzyme may also lead to disrupted mitophagy, which in turn has the capacity to dysregulate mitochondrial dynamics, and ultimately ATP production, further compromising energy generation.

Having reviewed the multiple mechanisms involved in driving the advent or exacerbation of chronic inflammation, oxidative stress and mitochondrial dysfunction, we will now consider possible therapeutic targets for the management of neuroprogressive and neurodegenerative diseases. Based on the mechanisms highlighted, it seems reasonable to suggest that molecules with the capacity to target the mechanisms driving ER stress and the UPR and to ameliorate the adverse downstream events following the activation of these pathways, would be desirable, and this consideration forms the basis of the approaches suggested below.

## Possible Neurotherapeutic Targets

### Melatonin

Recent evidence indicates that melatonin exerts a regulatory role in the process of mitophagy by stimulating the autophagic clearance of irreparably damaged mitochondria and increasing mitochondrial biogenesis, probably by a route involving the activation of AMPK and SIRT-1 [292–294]. Melatonin administration may also restore calcium homoeostasis, mitochondrial dynamics and mitochondrial permeability transition [295–298]. At least partly, those beneficial effects appear to be related to entry into the organelle, which may be facilitated by Glut-1 or peptide 1 and 2 transporter proteins located in the OMM [299, 300].

Moreover, melatonin may shift the pattern of mitochondrial dynamics towards a decrease in fission and an increase in fusion [296, 298, 301]. This activity has been demonstrated in a wide range of cell types [297, 301]. From a mechanistic perspective, the weight of evidence suggests that melatonin may attenuate the translocation of Fis1, Drp1 and Bax from the cytosol to mitochondria and may also upregulate mitochondrial fusion proteins Mfn1, Mfn2 and OPA1 [295, 300, 302, 303].

Melatonin supplementation also exerts multiple protective effects on mitochondria via a number of different mechanisms which may mitigate against the development of maladaptive processes within these organelles which initially stem from the upregulation of the UPR (i.e. ER stress). Such mechanisms include a reduction of mitochondrial oxidative stress [304, 305]; an increased efficiency of ATP production [306, 307]; a reduction in mitochondrial NOS expression [308, 309]; an amelioration of calcium dyshomoeostasis [310, 311]; the preservation of mitochondrial membrane potential [307, 312]; and a reduced release of cytochrome *c* into the cytosol accompanied by the inhibition of caspase-3 activity [313]. Several authors have demonstrated protective effects of melatonin supplementation against damage to mitochondria caused by a myriad of different insults including, but not limited to, sepsis [314, 315], ischaemia/reperfusion [316, 317] and challenge with neurotoxic compounds such as 1-methyl-4-phenylpyridinium ion (MPP^+^) [302], β-amyloid peptide (Aβ 25–35) [318, 319], 4-hydroxynonenal [320] and lipopolysaccharide [309] .

Melatonin therapy also inhibits the DNA binding activity and activation of NF-κB, with concomitant reductions in NLRP3 activity and the synthesis of pro-inflammatory cytokines [321–323]. These anti-inflammatory effects are considered to underpin the promising results obtained from studies investigating the use of melatonin in animal models of neurodegenerative diseases and are the motivation for an increased focus on the use of the molecule as a therapeutic agent targeting the pathogenesis and pathophysiology of diseases such as Alzheimer’s disease and Parkinson’s disease at doses ranging from 50 to 100 mg daily [305, 324]. In addition, it has been proposed that melatonin treatment could be useful for cognitive dysfunction associated with mood disorders [325]. However, evidence remains inconclusive [326].

### CoQ_10_

Converging preclinical and clinical evidence suggest that coenzyme Q_10_ (CoQ_10_) supplementation may offer therapeutic benefits in a range of neurodegenerative and neuroprogressive disorders, at least partly owing to its effects on ER stress and adverse downstream effects. For example, Yubero-Serrano et al. [327] reported that supplementation of CoQ_10_ in tandem with a Mediterranean diet effectively suppressed the expression of genes encoding proteins involved in the UPR. Furthermore, CoQ_10_ also appeared to exert a positive effect on mitochondrial dynamics by exerting a direct effect on ATP production [328], while this therapeutic target may also preserve the structure of mitochondrial cristae, with an accompanying increase in mitochondrial biogenesis [329]. In addition, CoQ_10_ may also restore endogenous anti-oxidants, such as vitamin E [330], and is also an essential player in enabling the optimal performance of the electron transport chain and stabilisation of the mitochondrial permeability transition pore [331] (reviewed by [332]).

There is also a considerable and increasing body of evidence demonstrating beneficial effects of CoQ_10_ on levels of pro-inflammatory cytokines and ROS, which may both act as a trigger of the UPR and be effectors of pathology following activation. For example, the effectiveness of CoQ_10_ supplementation at a daily dose of 500 mg for 12 weeks reduced inflammation and oxidative stress in a randomised, double-blind, placebo-controlled trial involving participants with relapsing-remitting multiple sclerosis [333, 334]. Controlled data demonstrating the ameliorative effects on inflammation and oxidative stress of CoQ_10_ supplementation also extend into other disease areas such as coronary artery disease, and there is some evidence that such benefits could be dose related [335]. In addition, evidence suggests that CoQ_10_ supplementation at doses up to 300 mg/day is safe and well tolerated [336, 337]. The former research team reported a significant reduction in cardiovascular mortality, over and above that seen in patients receiving standard treatment, in an elderly population of 445 patients supplemented with 200 mg of CoQ_10_ for 4 years [336], while the latter group of researchers found a significant reduction in cardiovascular morbidity and mortality in a population of 420 patients with chronic heart failure supplemented with 300 mg of CoQ_10_ for 2 years [337]. Human and animal studies have also demonstrated the potential for increased clinical benefit from the use of mitochondrial-targeted CoQ_10_ (MitoQ) where the active molecule is covalently attached to the lipophilic triphenylphosphonium cation [300, 338]. This mode of delivery allows levels of CoQ_10_ to accumulate within the mitochondrial matrix, reaching levels several hundred-fold higher than can be achieved via supplementation with the naked coenzyme [338, 339]. Human trials of the use of MitoQ in the treatment of neurodegenerative diseases have produced initial evidence of benefit with particularly encouraging results seen in Parkinson’s disease [340, 341]. In addition, CoQ_10_ could represent a novel therapeutic target for cognitive dysfunction associated with mood disorders [342], and a recent uncontrolled study found potential benefits for CoQ_10_ as a treatment for bipolar depression in late-life [343]. Clearly, the field awaits the design of large-scale and well-designed controlled trials testing CoQ_10_ as a therapeutic target for mood disorders.

### NAC

Animal and clinical studies have reported beneficial effects of *N*-acetylcysteine (NAC) supplementation on levels of ER stress [344–347]. For example, rats supplemented for 2 months with drinking water containing 600 mg NAC per litre displayed reduced levels of PDI and GRP78 compared with rats which did not receive NAC [345]. Similar findings indicate that NAC supplementation at 100 or 300 mg/kg for 20 weeks promote significant reductions in ROS levels [346, 347]. Moreover, NAC-related benefits upon ER stress appear to be dose-dependent, with 20 mmol/L of NAC being more effective than 10 mmol/L in reducing levels of GRP78 and ROS [348]. It should be noted, however, that NAC is a pleiotropic agent and several mechanisms other than direct effects on the UPR may also contribute to its therapeutic effects across neurodegenerative and neuroprogressive diseases [349, 350], while evidence suggests that adjunctive NAC treatment may mitigate cognitive dysfunction in a range of such disorders [351].

## Conclusions and Future Directions

This review indicates that pathways related to the UPR may reciprocally interact with immune-inflammatory, neuro-oxidative, neuro-nitrosative, as well as mitochondrial mechanisms, which are thought to play a major shared pathophysiological role across several neuroprogressive and neurodegenerative diseases. Therefore, the chronic upregulation of the UPR may interact with a range of cell death mechanisms underpinning neurodegeneration and neuroprogression [352] and hence represents a novel neurotherapeutic target.

Moreover, this review also opens relevant directions for further research. First, the involvement of mechanistic pathways related to the UPR in separate disorders deserves further investigation. Second, the extent to which effects upon the UPR could contribute to therapeutic benefits of novel therapeutic targets (for example, melatonin, CoQ_10_ and NAC) is a matter of ongoing research efforts. Lastly, the identification of patients who could benefit from therapies targeting ER stress pathways, taking in account the emerging framework of precision psychiatry [353], could represent a relevant road of research.
